# Evidence of Sex Differentiation Based on Morphological Traits During the Early Development Stage of Mud Crab *Scylla paramamosain*

**DOI:** 10.3389/fvets.2021.712942

**Published:** 2021-07-29

**Authors:** Wenxiao Cui, Shaobin Fang, Ligang Lv, Zhi Huang, Fei Lin, Qingyang Wu, Huaiping Zheng, Shengkang Li, Yueling Zhang, Mhd Ikhwanuddin, Hongyu Ma

**Affiliations:** ^1^Guangdong Provincial Key Laboratory of Marine Biotechnology, Shantou University, Shantou, China; ^2^Shantou University- Universiti Malaysia Terengganu, Joint Shellfish Research Laboratory, Shantou University, Shantou, China; ^3^Institute of Tropical Aquaculture and Fisheries, Universiti Malaysia Terengganu, Kuala Terengganu, Malaysia

**Keywords:** *Scylla paramamosain*, morphological differences, sex identification, development stages, biostatistical analysis

## Abstract

In order to uncover the sexual difference in morphology and how early they appear during the development stage of mud crab *Scylla paramamosain*, we measured, observed, and biostatistically analyzed morphological traits related to sex. For unveiling the morphological differences between sexes, morphological traits involving abdomen width (AW), carapace length (CL), and carapace width (CW) were first measured during the crablet development stage of *S. paramamosain* in the present study. The correlation analyses and path analyses exhibited that sexual dimorphism in the third abdomen width (AW3) and fourth abdomen width (AW4) could be used for sex identification from stage C VI (stage VI of crablet). Based on the stepwise discriminant analysis and standardized traits, a sex discriminant equation was constructed, which is capable for sex identification in crablets from stage C VI. Observations for secondary sexual traits and abdomen morphology (shape and pleopods) using a dissecting microscope or scanning electron microscope indicated that sexes are easily identified at stage C VIII according to the abdomen shape; meanwhile, at stage C II based on pleopod difference, and at stage C I by the presence or absence of gonopores. The findings in this study contribute greatly to the accuracy of sex identification of *S. paramamosain* during the early development stage, which promotes the understanding of the morphological differentiation mechanism of sex.

## Introduction

Sexual dimorphism deriving from sex differentiation of animals is always one of the critical propositions in life science, which makes life more complex and wonderful (1). The significant sexual differences in morphology and physiology are known as secondary sexual traits, which encompass a suite of traits from the external genitalia, to courtship behaviors, to any other sex-specific morphological, behavioral, or physiological traits (2, 3). These different traits allow each sex to better adapt to the environment and occupy a favorable ecological niche (4, 5). Sexual dimorphism is quite prevalent in the animal kingdom and has attracted researchers' attention (6–8). However, knowledge about the morphological difference between sexes of crablets remains lacking.

The mud crab *Scylla paramamosain* belongs to the genus *Scylla* and exhibits significant sexual dimorphism concerning growth rate, body size, and abdomen morphology (9). Apart from that, females have higher economic and nutritional values than males (10). As one of the traditional and prevalent marine crabs, the output of *S. paramamosain* has reached the leading position among marine commercial crabs in China (11, 12). Due to the demand fueled by increasing standards of living in the major consumer countries and preference for sexually mature female crabs with high nutritional value and better taste, the price of females is often two to three times higher than that of males (13). Therefore, it is significant for sex-biased breeding with the help of sex identification in morphology during the immature stage, and, monosexual breeding can be an effective solution to meet the ever-increasing market demand for females.

It is commonly accepted that the sexual differences of mud crab in morphology at the mature stage are mainly in the following aspects: (1) Four pairs of double-branched (biramous) pleopods are in the wide and rounded female abdomen. Contrary to the females, there is only one pair of uniramous gonopods in the narrow and straight male abdomen. Additionally, one pair of gonopores is in the third thoracic segment of the female, and no gonopore was found at the same position in the males (2) The females keep a faster growth rate and a bigger body size than the males except for the cheliped size (14). Apart from that, several studies have been implemented about morphological differences between the sexes of crabs. A clear sexual dimorphism in crab size was found in *Loxopagurus loxochelis*, with males reaching larger dimensions than females, mainly in terms of the major (i.e., left) chela size (15). In the male of *Eriocheir japonica*, ambulatory legs are relatively longer than females (16). Sexual dimorphism was evident in terms of claw size and coloration of the symbiotic pea crab *Austinixa aidae*. Males have larger claws than females, which gives them the favor in male–male competition. In addition, the body coloration of males was more similar to the sand grains of the beach than that of females (17). Furthermore, there were highly significant differences by sex in carapace length, carapace width, carapace height, and body weight for adult Chinese mitten crab (*Eriocheir sinensis*) (18). Besides, female adults had wider abdominal segments and a thicker body compared with males of the leucosiid crab *Pyrhila pisum* (19). Although several studies focused on the morphological differences in the mature crab were done, the study about the immature crab was still lacking. Especially in the *Scylla*, the sex of the immature mud crab is hard to identify, and the time of sexual differentiation in morphology has not been determined yet.

In crustaceans, females are identified by unique sexual morphological features dedicated to mating and breeding, which developed gradually before or at puberty but are hard to characterize in the early development stage (20). As far as we know, only several studies have been devoted exclusively to understand the specific time for sex identification of crablets by morphology. To find out the specific time for sex identification, Shi et al. (14) constructed a discriminant function equation based on growth traits of the juvenile mud crabs, which will provide great help for further research on the time of sex differentiation of the *S. paramamosain*. In the histological and morphological study of the crab *E. japonicus*, Lee et al. (21) found that sex differentiation first occurred in the gonoducts, and sexual dimorphism is visible in the abdomen at the fifth crab stage. In addition, there may be differences in some morphological traits between sexes during the development from crablet to adult in the Chinese mitten crab *Eriocheir sinensis* (18). As the secondary sexual characteristics, the female leucosiidae crab *Pyrhila pisum* gradually developed their abdomen during the juvenile stage and drastically enlarged their width and thickness by a puberty molt from juvenile to adult (19). Although some studies have been conducted, the information applied for sex identification in specific stages based on the morphological differences during the development of the *S. paramamosain* crablets is still lacking. Additionally, the previous study has revealed that the external morphological differentiation of *Scylla serrata* is earlier than gonadal differentiation (22).

In which stage can the mud crab be distinguished in males and females by sexual morphology? Also, at which stage does morphological sexual differentiation begin? In this study, we attempt to answer these questions in mud crab *S. paramamosain*. Hence, the morphological differences between sexes during the early development of mud crab *S. paramamosain* were studied to determine the differentiation time. Also, a discriminant function equation was constructed, by which the specific stages of sex differentiation in morphology is further determined. Exploring the critical period of morphological sex differentiation can provide the basis for sex control technology and the development of monosexual crab breeding techniques. It can also promote a deep understanding of the mechanism underlying the sexual differentiation of mud crab and other crabs.

## Materials and Methods

### Larvae and Crablet Culture and Sampling

The *S. paramamosain* larvae were hatched in circular fiberglass tanks (0.9 m in diameter, 1.0 m in height) at a local aquaculture farm (Guangdong Province, China), and then they were transferred to concrete rearing tanks (5.8 × 4.8 × 1.8 m). Culture conditions were ambient temperature, natural photoperiod, and salinity of ~28 ppt. The molting times were recorded, and the crablets at each stage were randomly sampled from a family. The molting and sampling time of the crablets at the eight stages are described in Table 1. Sampling time increased with molting time. A total of 72 crablets at each stage were sampled, of which 42 were for sex identification and morphological observation, and 30 were for observing by scanning electron microscope and dissecting microscope.

**Table 1:** The molting and sampling time at the different stages of the *Scylla paramamosain* crablets.

**Stage**	**C I**	**C II**	**C III**	**C IV**	**C V**	**C VI**	**C VII**	**C VIII**
Molting time		PJ3	PJ6	PJ10	PJ14	PJ19	PJ24	PJ31
Sampling time	PJ1	PJ3	PJ6	PJ10	PJ14	PJ19	PJ24	PJ31

*C I-VIII, crablet stage I-VIII; PJ, postjuvenile crab*.

### Morphological Trait Measurement and Observation

**Figure 1:**
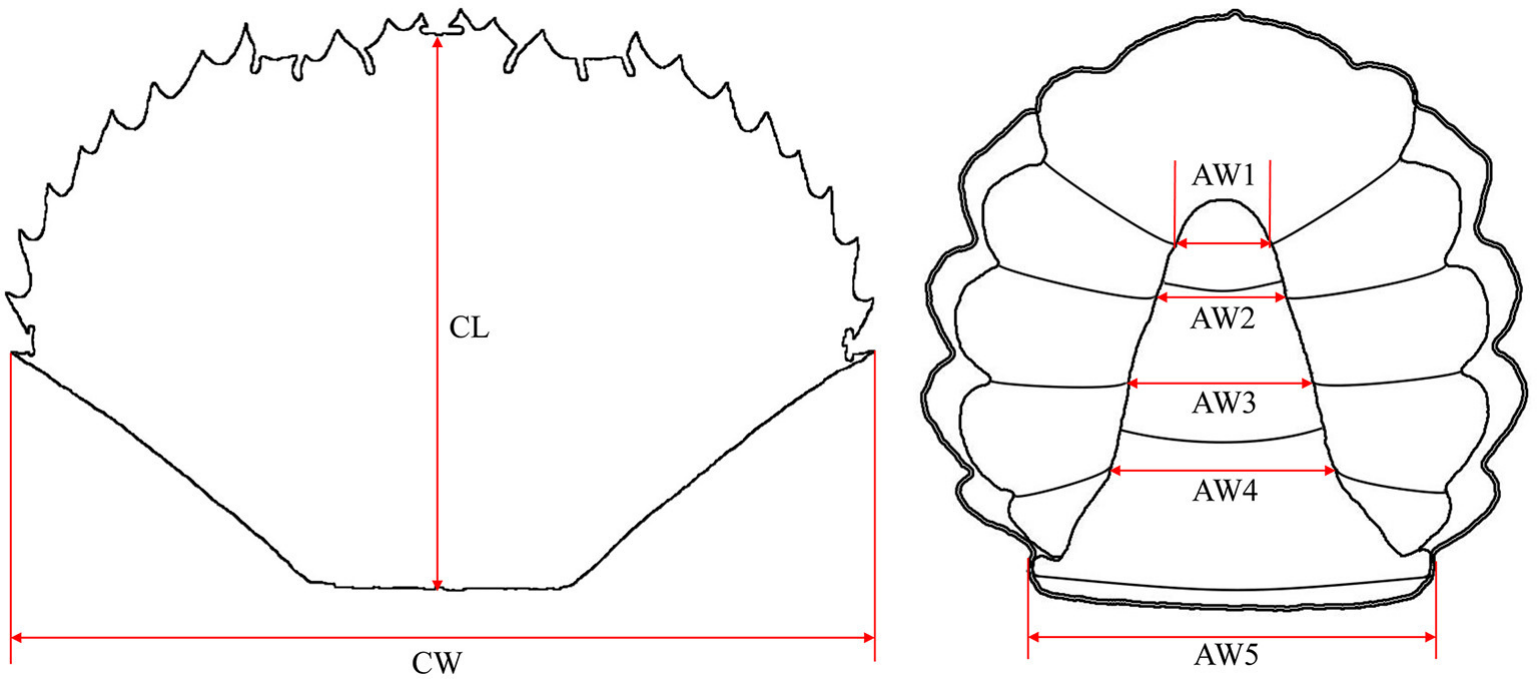
Measurement indexes of the *Scylla paramamosain* crablet. CL, carapace length; CW, carapace width; AW1, first abdomen width; AW2, second abdomen width; AW3, third abdomen width; AW4, fourth abdomen width; AW5, fifth abdomen width.

The first abdomen width (AW1), second abdomen width (AW2), third abdomen width (AW3), fourth abdomen width (AW4), and fifth abdomen width (AW5) were measured to the nearest 0.01 mm by standard vernier calipers (Figure 1). The carapace length (CL) and carapace width (CW) were measured using the methods of Gao et al. (23).

To study the abdominal differences between sexes in terms of morphology and secondary sex characters, we observed the abdomen shape, pleopods, and gonopore of the crablets using a dissecting microscope and scanning electron microscope.

### Identification of Genetic Sex

The genetic sex of the *S. paramamosain* crablets was identified following the method designed previously by our laboratory (10). A pair of female-specific primers, designed based on the sex-specific SNP markers, were used to amplify the sex-specific SNP locus, and the female-specific band was checked on agarose gel.

### Statistical Analysis

All data were analyzed with Microsoft Excel 2010 and IBM SPSS 20.0. Results are expressed as mean values with the standard error mean (SEM). Before the statistical analysis, data normality and homoscedasticity test were carried out using the Shapiro–Wilk test. One-way ANOVA and Tukey's multiple range tests were conducted to distinguish significant differences with statistical significance at *p* < 0.05 and high significance at *p* < 0.01. The coefficient of variation (CV) of each trait was estimated using the formula of CV = (standard deviation/mean) × 100%.

The correlation analysis between morphological traits with sex was performed using Pearson coefficient (two-tailed, *p*-value). The multiple regression equation was constructed, and the determination coefficient was calculated as described by Jiang et al. (24). For the construction of the discriminant function equation, the values of six traits (except CW) were normalized to CW and subsequently subjected to stepwise discriminant analysis by SPSS 20.0.

## Results

### Difference in Morphological Traits Between Sexes and Their Correlation With Sex

**Figure 2:**
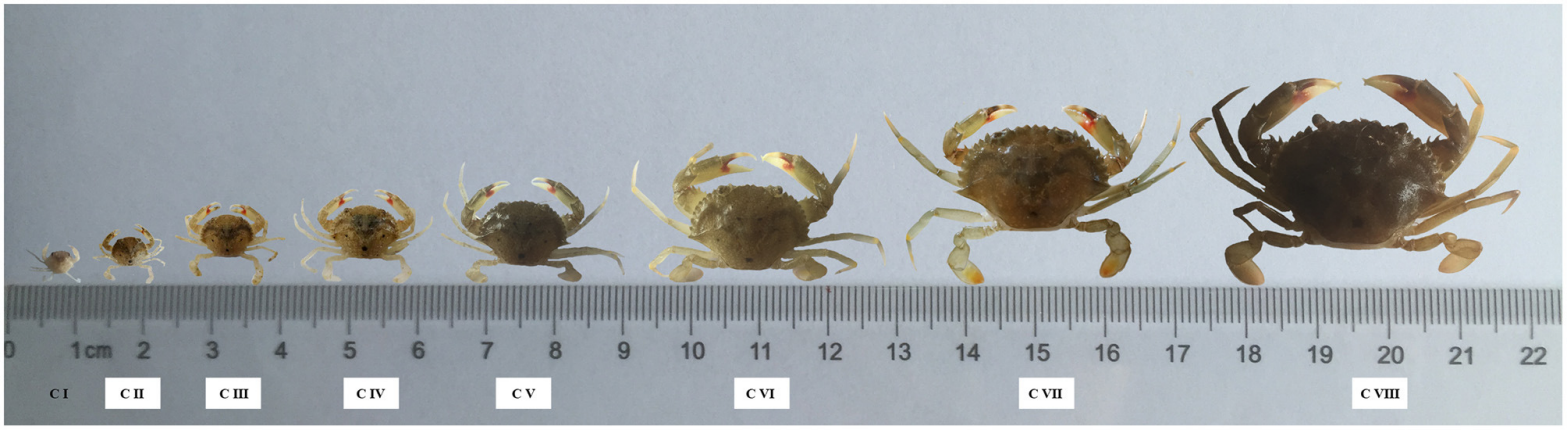
The size of the *S. paramamosain* at different stages. C I, C II, C III, C IV, C V, C VI, C VII, and C VIII represent crablets of stages I, II, III, IV, V, VI, VII, and VIII.

**Figure 3:**
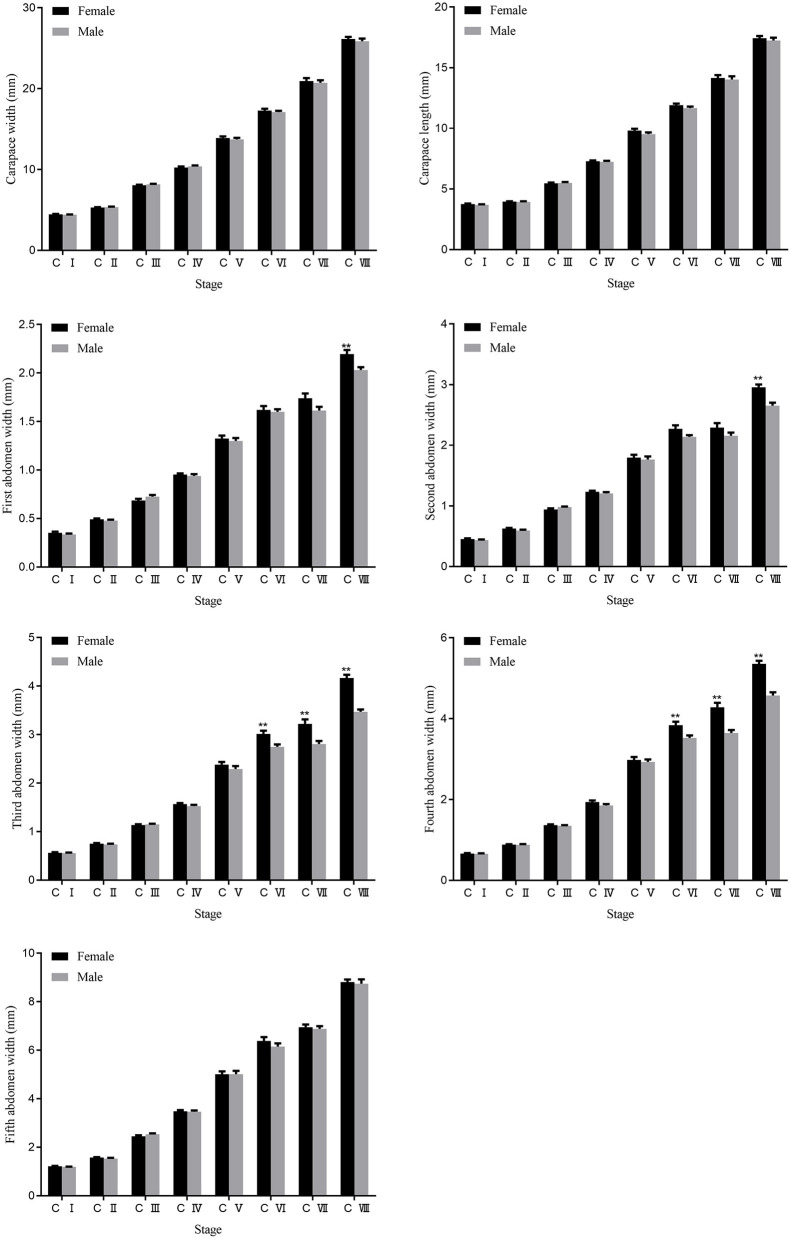
Changes and differences of seven traits between females and males during the development of the *S. paramamosain* crablets. ** represents significant differences at 0.01 level. C I, C II, C III, C IV, C V, C VI, C VII, and C VIII represent crablets of stages I, II, III, IV, V, VI, VII, and VIII. AW1, first abdomen width; AW2, second abdomen width; AW3, third abdomen width; AW4, fourth abdomen width; AW5, fifth abdomen width.

The crablets of the *S. paramamosain* were collected at the first day after each molting from stage C I to stage C VIII. The sample size and morphological traits at different stages are shown in Figure 2 and Supplementary Table 1. The change in these traits at the successive stages showed the morphological difference between sexes and morphological change during the developmental process. Of the seven morphological traits measured, all indexes increased gradually with the crablets growth (Figure 3). Apart from that, AW1 and AW2 values exhibited significant (*p* < 0.01) differences between sexes at stage C VIII. The AW3 and AW4 values of the females were significantly (*p* < 0.01) higher than those of the males from stage C VI. The values of CL, CW, and AW5 showed no differences between sexes.

The genetic sex of 336 crablets (42 crablets per stage) was successfully identified (Supplementary Figure 1 and Table 2). Although the number of male and female crablets was diverse at different stages, the female:male sex ratio of all crablets was 1:1. Besides, the sex ratio is close to or equal to 1:1 from stage C V to stage C VIII. Correlation analysis indicated that, from stage C I to C V, there was no significant correlation between the seven morphological traits and sex (Table 3). At stage C VI, AW3 and AW4 were highly and significantly correlated with sex (*p* < 0.01), and AW2 was significantly correlated with sex (*p* < 0.05). For stage C VII, both AW3 and AW4 were significantly correlated with sex with the correlation coefficient −0.514 and −0.609 (*p* < 0.05). At stage C VIII, AW1, AW2, AW3, and AW4 were highly and significantly correlated with sex (*p* < 0.01).

**Table 2:** Statistics of genetic sex at different development stages of *S. paramamosain* crablets.

**Stage**	**Number**	**Female**	**Male**	**Ratio (female/male)**
C I	42	19	23	0.83
C II	42	28	14	2
C III	42	22	20	1.1
C IV	42	17	25	0.68
C V	42	20	22	0.91
C VI	42	21	21	1
C VII	42	20	22	0.91
C VIII	42	21	21	1
Total	336	168	168	1

**Table 3:** Correlation coefficients between morphological traits and sex at the different development stages.

**Stage**	**CW**	**CL**	**AW1**	**AW2**	**AW3**	**AW4**	**AW5**
C I	−0.129	−0.097	−0.180	−0.180	−0.067	−0.067	−0.238
C II	0.096	−0.035	−0.167	−0.278	−0.132	−0.041	−0.201
C III	0.173	0.016	0.246	0.282	0.103	−0.111	0.219
C IV	0.199	−0.067	−0.098	−0.162	−0.196	−0.254	−0.054
C V	−0.062	−0.205	−0.093	−0.071	−0.170	−0.087	−0.001
C VI	−0.073	−0.261	−0.071	−0.306^*^	−0.442^**^	−0.426^**^	−0.170
C VII	−0.124	−0.067	−0.301	−0.248	−0.514^**^	−0.609^**^	−0.053
C VIII	−0.086	−0.100	−0.446^**^	−0.598^**^	−0.804^**^	−0.754^**^	−0.068

^**^ and ^*^ represent significant differences at 0.01 and 0.05 level, respectively.

### Determination of Coefficients and Path Coefficients

Multiple regression analysis and path analysis demonstrated that the seven morphological traits have no significant effects on sex identification from stage C I to stage C V, and the AW1, AW3, and AW4 have significant effects on sex identification from stage C VI to stage C VIII (*p* < 0.01) (Table 4). Additionally, AW4 has a more direct impact on sex identification compared with AW1 and AW3. Besides that, CW and AW4 at stage C VII, and CW and AW3 at stage C VIII exhibited significant effects on sex identification (*p* < 0.01). Moreover, the raising determination coefficient indicated that the effects of morphological traits on sex identification were increasing.

**Table 4:** Multiple stepwise regression analysis between the morphological traits and sex at three developmental stages.

**Stage**		**Trait**	**Coefficient**	**Direct effect**	**Indirect effect**	**Determination coefficient (R^**2**^)**	***P***	**Multiple regression equation**
C VI				AW1	AW3	0.334	0.000	
	AW1	−0.071	0.547	–	0.618			y = 1.815X_1_ – 1.410X_2_ + 2.637
	AW3	−0.442	−0.843	0.401	–			
C VII				CW	AW4	0.684	0.000	
	CW	−0.124	0.895	–	1.019			y = 0.295X_4_ – 1.240X_3_ + 0.256
	AW4	−0.609	−1.306	0.698	–			
C VIII				CW	AW3	0.765	0.000	
	CW	−0.086	0.391	–	0.477			y = 0.140X_4_ – 1.146X_2_ + 2.218
	AW3	−0.804	−0.992	0.188	–			

*X_1_, the first abdomen width; X_2_, the third abdomen width; X_3_, the fourth abdomen width; X_4_, carapace width; y, sex*.

### Construction of Discriminant Equations and Their Application

Given that the morphological traits were significantly related to sex only from stages C VI to C VIII, the values of morphological traits from these stages were used to construct discriminant equations. Five standardized morphological traits (AW1/CW, AW2/CW, AW3/CW, AW4/CW, and AW5/CW) were identified as contributors to Fischer's linear discriminant function of sex identification based on stepwise discriminant analysis. Discriminant equations were established as follows:

$$\begin{array}{l} F_{1}=138.964\text{ AW}1/\text{CW }-\;627.625\text{ AW}2/\text{CW}\\ \;+\;161.378\text{ AW}3/\text{CW }+\;583.418\text{ AW}4/\text{CW}\\ \;+\;283.938\text{ AW}5/\text{CW }-\;93.230;\\ F_{2}=277.494\text{ AW}1/\text{CW }-\;522.555\text{ AW}2/\text{CW}\\ \;+\;6.301\text{ AW}3/\text{CW }+\;456.279\text{ AW}4/\text{CW}\\ \;+\;323.078\text{ AW}5/\text{CW }-\;81.634. \end{array}$$

If F1 > F2, the candidate was estimated to be female; otherwise, it was male. The reliability assessment of the discriminant equations was conducted using 336 individuals from eight development stages (Table 5). By comparison with the genetic sex, more than 85% accuracy was detected from stages C VI to C VIII while low accuracy from stages C I to C V, which indicated that the discriminant equations are suitable only from stages C VI to C VIII.

**Table 5:** The accuracy of sex identification at different stages of the *S*. *paramamosain* crablets.

**Stage**	**Genetic sex**	**Number**	**Predicted** **sex**		**Identification accuracy (%)**	**Total discriminant accuracy (%)**
			**Female**	**Male**		
C VIII	Female	21	19	2	90.48	
	Male	21	2	19	90.48	90.48
C VII	Female	20	16	4	80	
	Male	22	2	20	90.91	85.71
C VI	Female	21	19	2	90.48	
	Male	21	4	17	80.95	85.71
C V	Female	20	15	5	75	
	Male	22	13	9	40.91	57.14
C IV	Female	21	4	17	19.05	
	Male	21	1	20	95.24	57.14
C III	Female	22	0	22	0	
	Male	20	0	20	100	47.62
C II	Female	24	0	24	0	
	Male	18	0	18	100	42.86
C I	Female	19	2	17	10.53	
	Male	23	4	19	22.61	50

### Sexual Difference of Secondary Sexual Traits and Abdomen Shape

**Figure 4:**
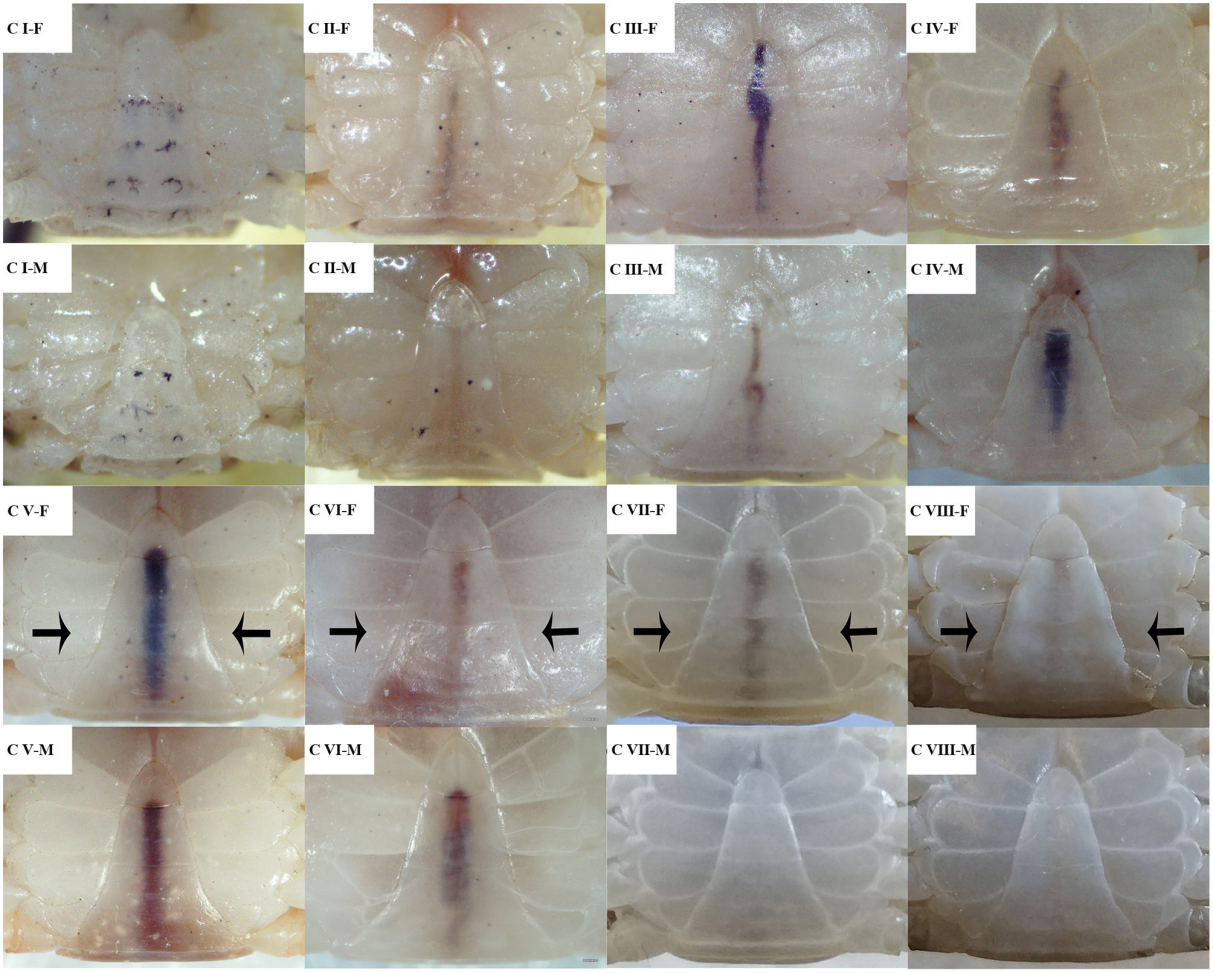
The external morphology of the abdomen at different development stages of male and female *S*. *paramamosain* crablets. F, female; M, male. The black arrows represent the segment where the shape was changed.

According to the genetic sex, the crablets were distinguished to male and female. Secondary sexual traits and their abdomen morphology were observed; as shown in Figure 4, the females shared the triangle-like shape of the abdomen with the males. However, the edges of the female abdomen start converting to a straight line at stage C VI; the counterpart of the male still maintains the original concave features. At stage C VIII, the edges of the female abdomen converted to a convex type line. With this feature, it is easy to distinguish sexes.

**Figure 5:**
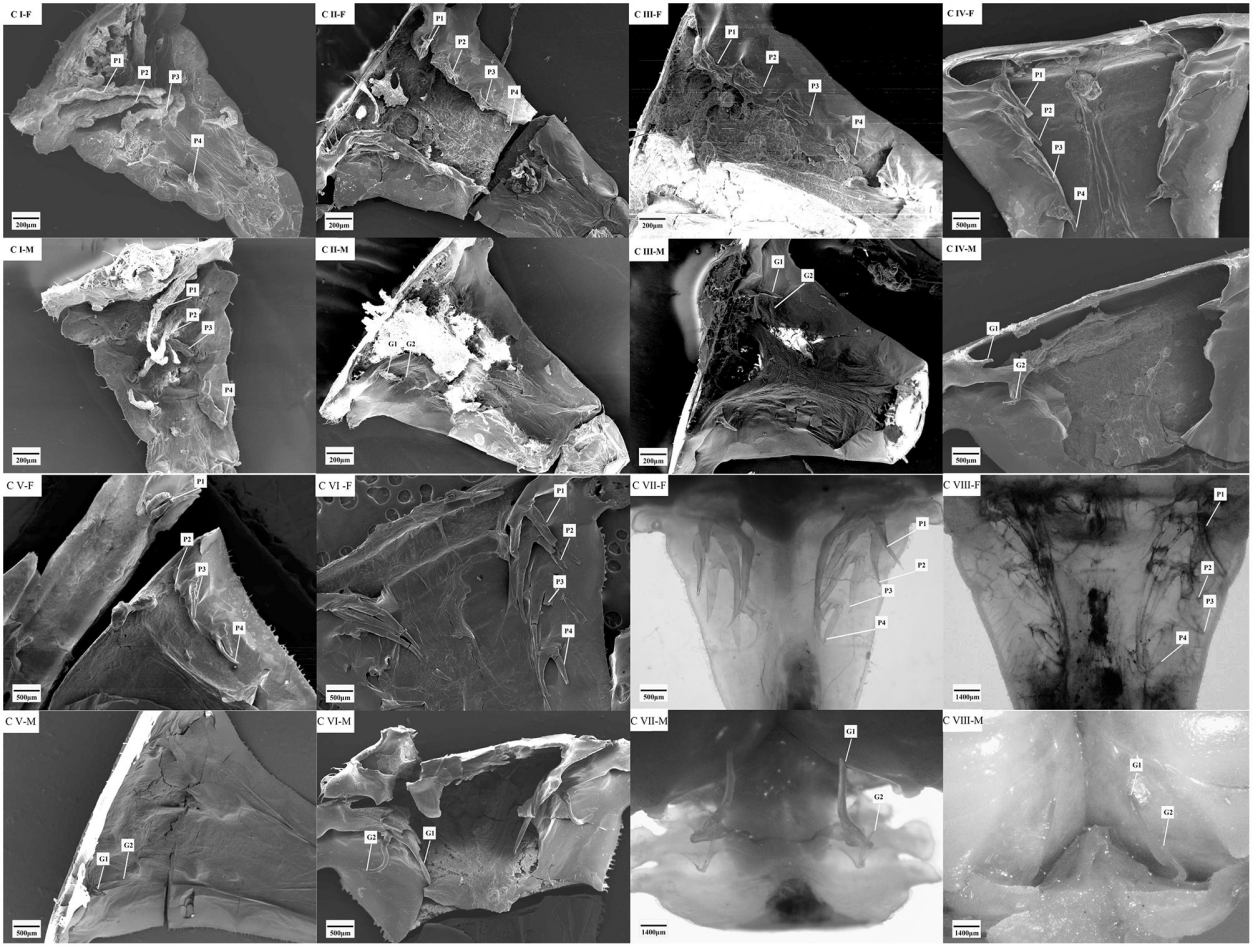
The secondary sexual characteristics of the *S. paramamosain* crablets. C I, C II, C III, C IV, C V, C VI, C VII, and C VIII represent crablets of stages I, II, III, IV, V, VI, VII, and VIII. F, female; M, male; G1 and G2 represent gonopods 1 and 2; P1, P2, P3, and P4 represent pleopods 1, 2, 3, and 4.

Transformation of pleopods during the development was observed with the scanning electron microscope (Figure 5). At stage C I, the female crablet has four pairs of slender uniramous pleopods that vary in length (P1 > P2 > P3 > P4), as well as the males. Secondary sexual characteristics begin to appear at stage C II, in which the slender uniramous pleopods of the female crablets were shortened and transformed to the biramous. In male crablets, P1 and P2 were shortened and metamorphosed into clublike gonopods, while P3 and P4 degenerated and disappeared at stage C II. In the developmental stages from stages C III to C VIII, the pleopods were ever-growing with a constant length ratio in both sexes (P1 > P2 > P3 > P4 for the females and P1 > P2 for the males), and the P2 of the males was metamorphosed finally into the mating organ.

**Figure 6:**
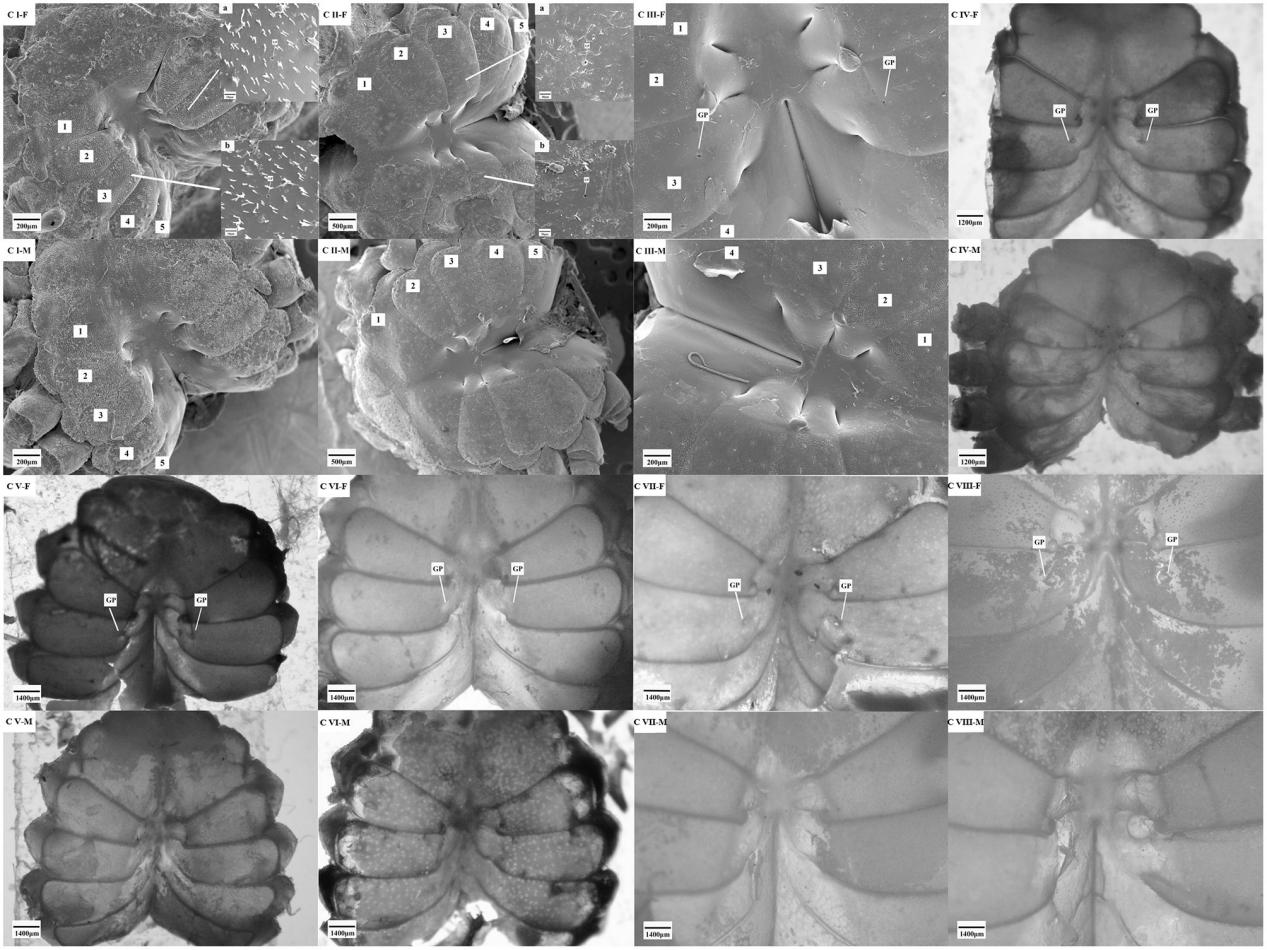
The gonopore characteristics of the *S. paramamosain* crablets. F, female; M, male; GP, gonopores. C I, C II, C III, C IV, C V, C VI, C VII, and C VIII represent crablets of stages I, II, III, IV, V, VI, VII, and VIII. 1–5, first–fifth thoracic segment.

Through the observation of the secondary sex character, a comparative analysis of the gonopores was carried out (Figure 6). The results showed that a pair of gonopores, which were located on the third thoracic segments and near the central axis, were enclosed by the thin cuticle at all stages (from stages C I to C VIII) of the females. Conversely, no gonopore was found at the same position in the males.

## Discussion

In the *S. paramamosain*, the adult females possess a wider and more rounded abdomen, which covers four pairs of biramous pleopods, compared with the adult males. This pattern of sexual dimorphism in abdomen morphology is in accord with previous observations of other *Scylla* crabs (25, 26). However, the first sign of sexual differentiation in morphology, represented by the beginning of abdominal morphological differentiation and development, is still unclear. The specific time of the sex differentiation in abdominal morphology was herein explored based on the observation and measurement of morphological traits of crablets. The results will provide a simple and effective method to distinguish the sex of mud crab in early development from the external morphology. First, the abdominal morphology of the males and the females was compared and analyzed from stages C I to C VIII. The results demonstrated that the sex of crablets could be first distinguished according to AW3 and AW4 from stage C VI in addition to AW1 and AW2 from stage C VIII. By quantifying the abdominal morphology of crablets, the correlation between abdominal morphology and sex was analyzed by one-way ANOVA. The abdomens of females were significantly rounder than that of males as they grow, with the sexual differences of AW3 and AW4 from stage VI, suggesting that the two morphological traits are significantly correlated with sex (*p* < 0.05). The finding in the present study is consistent with the previous research to a certain extent, in which the abdominal segments corresponding to AW3 and AW4 were applied to distinguish sexes (27). Significant correlations were found between sex and AW1, AW2, AW3, and AW4, but AW3 and AW4 are the most appropriate morphological traits for sex identification based on the comparison between sexes and one-way ANOVA. Besides, traits AW1, AW3, and AW4 have a significant direct effect on sex identification, which were found from stage C VI to C VIII based on path analysis, suggesting that it is feasible to discriminate sex with the resort to these abdominal morphologies from stage C VI. Our findings here are in accordance with the report by Flores et al. (28), in which the morphological difference of the abdomen can be used to determine sex when the crablets grow up to a certain stage. Combined with the results of path analysis, stage C VI, which could be adopted to distinguish the sex of crablets, was further determined.

To ensure the high accuracy of sex identification, the data-based discriminant equations and path analysis were performed to derive the predicting models for estimating the magnitude and significance of causal connections between traits and sex identification (14, 29). In the present study, the sex discriminant equations, applicable to the crablets from stage C VI, were constructed based on standardized morphological traits. Path analysis indicated that the morphological traits AW3 and AW4 have direct impacts on sex identification. In the earlier studies, morphological traits were considered as the key factors affecting body weight in the *S. paramamosain* crablets and were standardized to establish a reliable and immediate equation for sex identification (14, 24). In conclusion, our study indicated that the morphological traits AW3 and AW4 could be used to identify the sex of crablets from stage C VI.

The traits directly related to reproduction are frequently observed in many species and have been used as an effective method for sexing (30). Gonopore, a reproductive aperture or pore of certain insects and worms, is the reproductive organ that is the remarkable evidence for the sex identification of mud crab in addition to gonopods (31). The results of this study demonstrated that the sex of crablets would be distinguished based on the presence of gonopores or not at stage C I and gonopods at stage C II. This finding is consistent with the research on other decapods. Lee et al. (21) reported that in the crab *Eriocheir japonicus*, the gonopores of the females were visible at stage C I, while the different gonopods in sexes were discerned at stage C III. Sex identification can be implemented at stage C II when the sex differences regarding gonopores and gonopods were observed on *Callinectes sapidus, Rhizocephalan harrisii*, and *Menippe mercenaria* (32). In summary, the differentiation time of external sexual characteristics of decapods is diverse among different species (33).

Although it happened at different stages, the sex-related variation in morphological traits on crablets caters for the need for reproduction of the females who needs to carry their eggs beneath their abdomens until hatching (34). Generally, the morphological traits AW3 and AW4 can be regarded as the indicators for sex determination from stage C VI. The sex of crablets could be distinguished based on the presence of gonopores or not at stage C I and gonopod traits at stage C II using a dissecting microscope or scanning electron microscope. Hence, under the circumstance of keeping unstressed and uninjured aquatic animals, the combination of observation and biostatistical analysis will be a powerful method to examine the relationships between a dependent variable (like sex or bodyweight) and two or more independent variables (morphological characteristics).

## Conclusion

In the present study, seven morphological traits were measured during the crablet development stage of the *S. paramamosain* to research the sexual difference in morphology and morphological change. The correlation analyses between sex and morphological traits indicated that AW3 and AW4 could be implemented to identify sex from stage C VI. A sex discriminant equation was constructed based on the stepwise discriminant analysis and standardized traits, providing a possibility in sex identification of crablets from stage C VI. Path analysis showed that sex identification can be put into practice following the arisen difference of AW3 and AW4 between sexes from stage C VI. Sexes are easily distinguished at stage C VIII according to the abdomen shape, at stage C II based on pleopod difference, and at stage C I based on the presence of gonopores or not.

## Data Availability Statement

The original contributions presented in the study are included in the article/Supplementary Material, further inquiries can be directed to the corresponding author/s.

## Author Contributions

WC participated in the experimental design, investigations, data analyses, interpretation, and drafted the manuscript. SF participated in the experimental design and data analysis. LL, ZH, FL, and QW participated in the investigations. HZ, SL, YZ, and MI participated in the manuscript modification. HM participated in the experimental design and provided all materials and reagents. All authors contributed to the article and approved the submitted version.

## Conflict of Interest

The authors declare that the research was conducted in the absence of any commercial or financial relationships that could be construed as a potential conflict of interest.

## Publisher's Note

All claims expressed in this article are solely those of the authors and do not necessarily represent those of their affiliated organizations, or those of the publisher, the editors and the reviewers. Any product that may be evaluated in this article, or claim that may be made by its manufacturer, is not guaranteed or endorsed by the publisher.

## Supplementary Material

Supplementary Figure 1 Genetic sex identification of some individuals at the different development stages of the *S. paramamosain* crablets. M, maker, C I, C II, C III, C IV, C V, C VI, C VII, and C VIII represent crablet of stage I, II, III, IV, V, VI, VII, and VIII.

Supplementary Table 1 Statistics of growth traits in different development stage of the juvenile *S. paramamosain*.
