# Supplementary material for: Evidence of Sex Differentiation Based on Morphological Traits During the Early Development Stage of Mud Crab Scylla paramamosain
Source: Front Vet Sci. 2021 Jul 29;8:712942. doi: 10.3389/fvets.2021.712942 (PMC8358326; doi:10.3389/fvets.2021.712942)
Supplement: Supplementary Figure 1 — Genetic sex identification of some individuals at the different development stages of the S. paramamosain crablets. M, maker, C I, C II, C III, C IV, C V, C VI, C VII, and C VIII represent crablet of stage I, II, III, IV, V, VI, VII, and VIII. [file Table_1.DOCX]

**Table S1.** Statistics of growth traits in different development stage of the juvenile *S. paramamosain.*

| Stage | C Ⅰ | C Ⅱ | C Ⅲ | C Ⅳ | C Ⅴ | C Ⅵ | C Ⅶ | C Ⅷ |
| --- | --- | --- | --- | --- | --- | --- | --- | --- |
| MN | 42 | 42 | 42 | 42 | 42 | 42 | 42 | 42 |
| ACW (mm)  (Min-Max) | 4.421 ± 0.185  (4.20-4.80) | 5.310 ± 0.268  (4.80-5.90) | 8.102 ± 0.321  (7.50-8.60) | 10.270 ± 0.438  9.41-11.03 | 13.699 ± 0.778  (11.19-14.88) | 17.187 ± 0.834  (15.82-18.65) | 20.845 ± 1.533  (18.85-23.40) | 26.024 ± 1.412  (23.73-30.01) |
| ACL (mm)  (Min-Max) | 3.676 ± 0.175  (3.40-4.00) | 3.924 ± 0.179  (3.50-4.30) | 5.495 ± 0.289  (4.50-5.90) | 7.240 ± 0.318  (6.60-7.95) | 9.678 ± 0.572  (7.57-10.58) | 11.827 ± 0.567  (10.93-13.48) | 14.092 ± 1.161  (12.08-15.95) | 17.337 ± 0.903  (15.55-19.52) |
| AW(g)  (Min-Max) | 0.019 ± 0.003  (0.014-0.019) | 0.040 ± 0.005  (0.030-0.048) | 0.066 ± 0.007  (0.051-0.080) | 0.173 ± 0.035  (0.121-0.218) | 0.391 ± 0.057  (0.279-0.498) | 0.738 ± 0.115  (0.555-0.959) | 1.708 ± 0.297  (1.339-2.199) | 3.663 ± 0.670  (2.400-4.530) |

MN: measured number; ACW: average carapace width; ACL: average carapace length; AW: average weight; Min: minimum value; Max: maximum value. Due to the too slight weight, the individual weight of C Ⅰ crablets was obtained by which the total weight of the population divided by its number.
