# Supplementary figures and images for: Evidence of Sex Differentiation Based on Morphological Traits During the Early Development Stage of Mud Crab Scylla paramamosain
Source: Front Vet Sci. 2021 Jul 29;8:712942. doi: 10.3389/fvets.2021.712942 (PMC8358326; doi:10.3389/fvets.2021.712942)

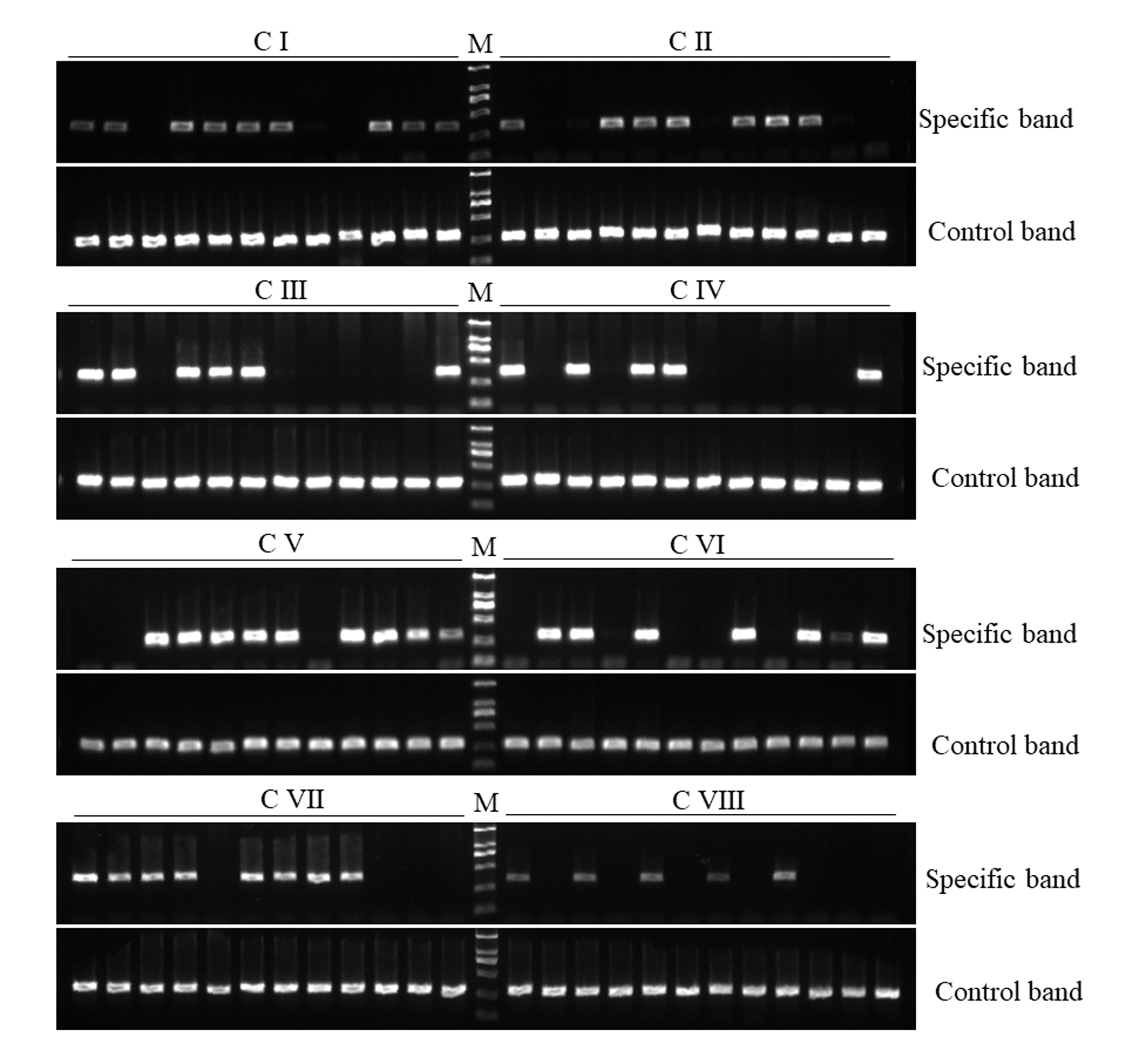

Supplement: Supplementary Table 1 — Statistics of growth traits in different development stage of the juvenile S. paramamosain. [file Image_1.TIF]
